# Prevalence of anxiety and depression among students and employees during the COVID-19 pandemic

**DOI:** 10.1192/j.eurpsy.2022.784

**Published:** 2022-09-01

**Authors:** T. Ionescu, E. Minecan, S. Zaharia, B. Fetecau, C. Tudose

**Affiliations:** 1 University of Medicine and Pharmacy Carol Davila, Neuroscience 6, Bucharest, Romania; 2 Alexandru Obregia Clinical Hospital of Psychiatry, Child And Adolescent Psychiatry, Bucharest, Romania; 3 University of Medicine and Pharmacy Carol Davila, Cardiology, Bucharest, Romania

**Keywords:** Covid-19, Depression, pandemic, Anxiety

## Abstract

**Introduction:**

Facing the COVID-19 pandemic, individuals are experiencing severe mental distress. Following social distancing and economic insecurity, significant increases in mental health concerns have developed.

**Objectives:**

The aims of this study was to report the levels of depressive and anxiety within active population in Romania, and to identify possible risk and protective factors for mental health.

**Methods:**

Data collection occurred between February-March 2021. The online survey included questions regarding socio-demographic characteristics and Hospital Anxiety and Depression Scale (HADS). 620 responses were validated (331 students and 289 workers).

**Results:**

Among active population, risk of anxiety symptoms is lower in those who already were infected with SARS-Cov2 (p=0.026, df=2, Phi=0.109), while positive screening for anxiety or depression in this study was statistically significant associated with younger age (p=0.026, df=4, Phi=0.134) and female gender (p=<0.001, df=2, Phi=0.166). Even though anxiety and depression scores are similar among students and employees, there are different aspects regarding symptomatology between these two groups. Students have experienced more frequently tendency to worry and sudden feelings of panic (p=0.004, df=3, Phi=0.146). Also, their ability to laugh and see the funny side of things is affected (p=0.019, df=3, Phi=0.127) and they feel less enthusiasm about future (p=0.001, df=3, Phi=0.159). Participants living with someone else scored lower on anxiety and depression subscales and those are not influenced by the person with whom they are cohabitating or residing (p=0.020, df=3, Phi=0.138).

**Conclusions:**

Findings from the current study offer initial insights into the rates of anxiety and depression within active population in Romania, one year after the onset of the COVID-19 pandemic.

**Disclosure:**

No significant relationships.

